# Fracture of the Patella

**Published:** 1877-07

**Authors:** M. E. Poynter


Fracture of the Patella—A New and Simple Device for Dressing.—*By M. E. Poynter, M.D.*—A great many modes have been adopted, and instruments invented, for the cure of this rather rare and very intractable accident. None of these have given entire satisfaction. Many are very complicated and costly, whilst others inflict great suffering upon the patient. Unsatisfactory results almost always follow the treatment, and ligamentous union is about all the surgeon expects, with separation of the fragments varying from a fourth of an inch to three inches.

Simplicity of construction, with easy adaptation to the ends sought, together with effective results, are desiderata in all surgical dressings. The nearer a surgeon can come to making and fitting his dressings for fractures at the bedside, so as to meet the requirements of any given case, the more nearly will he approximate the end desired, viz., the cure of his patient. This often requires considerable mechanical skill, and that a surgeon should possess.

By reference to the current text-books, it will be seen that the complicated apparatus recommended by Druit, and the inquisitorial hooks of Malgaigne, have been abandoned by the later authors, and a reliance is placed upon variously disposed bandages and adhesive strips, with a long, straight back splint and elevated foot piece—all requiring confinement to bed from six weeks to three months. The ring approached more nearly to perfection, but it has some disadvantages—as difficulty in fitting accurately, the cutting off of circulation, and trouble to keep in place. The instrument herewith presented obviates many or all these difficulties, and is so easy of construction that any surgeon can make it with his pocket knife in a few minutes, and adapt it properly at the bedside of his patient. The only materials wanted are an ordinary cigar-box, or a couple of shingles, and a straight piece of board 1½ or 2 feet long. It will be understood, of course, that this is intended exclusively for transverse fractures, and the great object to be attained is approximation of the fragments and retaining them in position until union can occur.

Procure the bottom and top pieces of the cigar-box, and split two pieces two inches wide, and hollow out one end of each to a semi-circle (only a little shallower). Trim the board back of this somewhat into the shape of the handle of a paddle; take a few nails from the cigar-box, and fasten close to the hollow end of each of these pieces a block of wood an inch long and half an inch square. Groove out the block a little on the end, away from the semi-circle, for the purpose of retaining a piece of cord in the way of a pulley. You will next procure a straight board to reach from the middle of the thigh to the middle of the calf, a roller and some lint or old muslin, and a piece of stout cord, and you will have all you will need.

First apply a thickness or two of lint or muslin to the under surface of each of your short splints, and apply one above and the other below the fractured patella, permitting the semi-lunar end to grasp its part of the bone accurately; line the long splint, and apply on back of limb; let an assistant hold them so, whilst you apply a roller from the toes to the middle of the thigh, observing as you do so that the short splints are in line on top of the limb. You will then be ready to bring the fragments together, which you will have no trouble whatever in doing, by taking a couple of turns of a stout cord around the little blocks, and drawing gently but firmly on them. Tie the cord, and you are done. You will perceive that the bones are held firmly in apposition and cannot readily be displaced. If you have a compound fracture, or much bruising of the soft parts, your dressing has left the whole surface open for treatment, and the straight splint allows no pressure upon the popliteal vessels, at the same time giving you extension and a firm basis for fixing the short splints. Put your patient on his feet with a crutch, and send him out of doors; he need not be kept confined an hour.

I have used this dressing several times, just as given above, and with the happiest results. If this bone can have ossific union, I think I have attained it, as I have been unable to detect either separation or ligament. In fact, this simple affair has been more satisfactory in results than any fracture dressing of any kind I have ever seen.—*Clinic.*

